# Monitoring of Plant Protein Post-translational Modifications Using Targeted Proteomics

**DOI:** 10.3389/fpls.2018.01168

**Published:** 2018-08-17

**Authors:** Borjana Arsova, Michelle Watt, Björn Usadel

**Affiliations:** ^1^Institut für Bio- und Geowissenschaften, IBG-2–Plant Sciences, Forschungszentrum Jülich, Jülich, Germany; ^2^IBMG: Institute for Biology I, RWTH Aachen University, Aachen, Germany

**Keywords:** targeted proteomics, plants, Arabidopsis, Berley, SRM/MRM, post translational modification, phosphorylation, ubiquitination

## Abstract

Protein post-translational modifications (PTMs) are among the fastest and earliest of plant responses to changes in the environment, making the mechanisms and dynamics of PTMs an important area of plant science. One of the most studied PTMs is protein phosphorylation. This review summarizes the use of targeted proteomics for the elucidation of the biological functioning of plant PTMs, and focuses primarily on phosphorylation. Since phosphorylated peptides have a low abundance, usually complex enrichment protocols are required for their research. Initial identification is usually performed with discovery phosphoproteomics, using high sensitivity mass spectrometers, where as many phosphopeptides are measured as possible. Once a PTM site is identified, biological characterization can be addressed with targeted proteomics. In targeted proteomics, Selected/Multiple Reaction Monitoring (S/MRM) is traditionally coupled to simple, standard protein digestion protocols, often omitting the enrichment step, and relying on triple-quadruple mass spectrometer. The use of synthetic peptides as internal standards allows accurate identification, avoiding cross-reactivity typical for some antibody based approaches. Importantly, internal standards allow absolute peptide quantitation, reported down to 0.1 femtomoles, also useful for determination of phospho-site occupancy. S/MRM is advantageous in situations where monitoring and diagnostics of peptide PTM status is needed for many samples, as it has faster sample processing times, higher throughput than other approaches, and excellent quantitation and reproducibility. Furthermore, the number of publicly available data-bases with plant PTM discovery data is growing, facilitating selection of modified peptides and design of targeted proteomics workflows. Recent instrument developments result in faster scanning times, inclusion of ion-trap instruments leading to parallel reaction monitoring- which further facilitates S/MRM experimental design. Finally, recent combination of data independent and data dependent spectra acquisition means that in addition to anticipated targeted data, spectra can now be queried for unanticipated information. The potential for future applications in plant biology is outlined.

## Phosphorylation: importance in regulation of plant processes and most common techniques for mass-spectrometric analysis

Plant post-translational modifications (PTMs) have been implicated in the regulation of a number of regulatory and metabolic processes. Among them, protein phosphorylation is the most studied PTM to date. As a product of enzymatic activity, it was first discovered at the beginning of the twentieth century (Levene and Alsberg, 1906; Burnett and Kennedy, 1954). It is controlled by a fine balance between kinases and phosphatases (Schulze, 2010). Indeed between 4 and 5% of the Arabidopsis genome encodes various kinases, which is almost double from mammals (Schulze, 2010; Zulawski et al., 2014).

Phosphorylation appears to be involved in regulating most of the metabolic and physiological pathways in plants, including: defense (Jones et al., 2006; Nühse et al., 2007), RNA metabolism (de la Fuente van Bentem et al., 2006), carbon metabolism (Wu et al., 2014), and root growth (Zhang et al., 2013, 2016).

Today, mass spectrometry (MS) is used routinely to analyze large phospho-proteomics datasets in an untargeted manner (Niittylä et al., 2007; Engelsberger and Schulze, 2012; Hoehenwarter et al., 2013; Silva-Sanchez et al., 2015; Nukarinen et al., 2016; Pi et al., 2018). These, are generated after complicated, multistep enrichment protocols like Immobilized metal affinity chromatography (IMAC), Titanium dioxide (TiO_2_), and Cerium(IV) oxide (CrO_2_) (Thingholm et al., 2008; Schulze, 2010; Qiao et al., 2012; Silva-Sanchez et al., 2015). The critical step in MS analysis involves detection of the loss of phosphate (neutral loss 98 kDa) from serine (Ser), threonine (Thr), and tyrosine (Tyr) in MS3. This occurs after the peptide has been fragmented to its amino acids. Phosphoproteomics data from these untargeted experiments are hosted in publicly accessible databases (Nühse et al., 2004; Gao et al., 2009; Durek et al., 2010).

Characterisation of the biological importance of specifiic phospho-sites was commonly done by biochemical and targeted molecular biology approaches like phospho-site substitution experiments (Budde and Chollet, 1986; Huber et al., 2002; Liu and Tsay, 2003; Lillo et al., 2004; Lanquar et al., 2009; Krouk et al., 2010; Dissmeyer and Schnittger, 2011; O'Leary et al., 2011). However, publicly available phospho-peptide information, now enables wide audiences to undertake physiological characterization of selected phosphoproteins by targeted proteomics.

The advantages of targeted proteomics over e.g., immunoassays to study phosphorylation include: more proteins monitored in a single run, less time invested in assay development, no cross-reactivity, and no need to raise antibodies against proteins with PTMs. In comparison to ^32^P radio-assays-there are lower safety risks, due to the use of stable isotopes. After the initial SRM assay is developed, the speed and throughput for processing samples significantly increases.

## Overview of targeted proteomics and considerations for study of phosphopeptides

While, in untargeted proteomics as many peptides are measured as possible (Schulze and Usadel, 2010), a characteristic of targeted proteomics is that the MS is tuned to only measure selected peptides from proteins of interest, commonly up to a few hundred (Borràs and Sabidó, 2017). Less peptides usually translates to shorter chromatography runs, resulting in higher sample throughput. The peptides must be unique to the protein (proteotypic), to allow unambiguous identification. Additionally, Osinalde et al. (2017) addresses important considerations about the chemical properties of phosphopeptides including phospho-isomers (peptide with same sequence but various possible phosphorylation sites).

Traditionally the MS- a triple quadrupole, is tuned to detect the whole peptide and the products of its fragmentation, after an *in silico* analysis. A triple quadrupole instrument uses the first quadruple to select the mass and charge of the whole peptide as it elutes from a liquid chromatography column (precursor ion, MS1), also recording the intensity of the signal over time. The second quadruple is used for collision induced fragmentation, and the third to record the intensity of previously specified peptide fragments for precise peptide identification and quantification (product ions, MS2) (Lange et al., 2008). The combination of precursor and product ions is called transitions and is unique to each selected peptide. If quantitation is performed based on the peptide signal in MS1 the technique is known as Selected Ion monitoring (SIM), while if quantitation is performed on the peptide fragments recorded in the third quadrupole—it is called Selected or Multiple Reaction Monitoring (S/MRM), and both are summarized elsewhere (Borràs and Sabidó, 2017). Synthetic peptides with amino acids containing stable isotopes of naturally low abundance, are spiked in as internal standards. In context of PTMs a modified and non-modified synthetic version of the peptide are included, whose elution times and potentially transitions will vary. The standards, which have same chemical properties as the native peptides—will elute from the liquid chromatograph with the native form and create similar fragment spectrum, but synthetic peptides and fragments can be identified by their different mass. Thus, the intensity of their signal serves for absolute quantitation, by comparison to the native peptide.

Traditional S/MRM is a technique where only a few peptide transitions are measured for each peptide (Lange et al., 2008; Elschenbroich and Kislinger, 2011). In contrast, parallel reaction monitoring (PRM) replaces the last quadruple with an Orbitrap-type instrument and monitors all fragments originating from the selected peptide, thus facilitating the initial method development (Peterson et al., 2012; Tang et al., 2014; Yang and Li, 2015; Bourmaud et al., 2016). A comparison between PRM and SRM showed comparable linearity, precision and dynamic range (Ronsein et al., 2015). Detection limits in PRM as low as 100 attomoles has been reported (Majovsky et al., 2014). A promising recent development is an approach that combines data independent and data dependent acquisition (i.e., Sequential Windowed Acquisition of All Theoretical Fragment Ion Mass Spectra: SWATH) where all peptides in a sample are fragmented and recorded, allowing the scientist to data-mine not only for targeted transitions but for novel information. In context of this review this translates to the potential for identification of novel PTM sites (Doerr, 2012; Aebersold et al., 2016; Keller et al., 2016; Osinalde et al., 2017). Notably, a cheaper alternative to create large numbers of internal standards for quantitation, is the use of amino acid labeled recombinant synthetic proteins, composed of concatenated prototypic peptides (QconCAT) separated by tryptic sites. This approach has been adapted to study the stoichiometry of protein phosphorylation (Johnson et al., 2009; Pertl-Obermeyer et al., 2016).

## Design of SRM experiments for targeting protein phosphorylation

Since publication of the first Arabidopsis genome in 2000 (Arabidopsis Genome, 2000), basic plant science has progressed largely with model organisms to take advantage of their molecular tool-boxes and mutant collections, which are costly to develop for individual species. Notably, application of the CRISPR technology may partially change this trend for crop plants (Song et al., 2016). Nevertheless, progress in next generation sequencing technologies brings a steady rise in available genomes and transcriptomes (Bolger et al., 2017), which means we can build on existing knowledge through the use of conserved phosphorylation sites (Schulze et al., 2012). For example, in a comparison of phosphorylation sites between Arabidopsis and Rice, over 50% of the proteins that possessed orthologues were found to have an orthologous phosphoprotein in the other species, and about half were phosphorylated at equivalent sites (Nakagami et al., 2010). This means that a large part of information and methods can be carried across species. Although the phosphorylation prediction tool, Musite, can be used (Yao et al., 2012), an experimental confirmation is often necessary.

**Figure 1:**
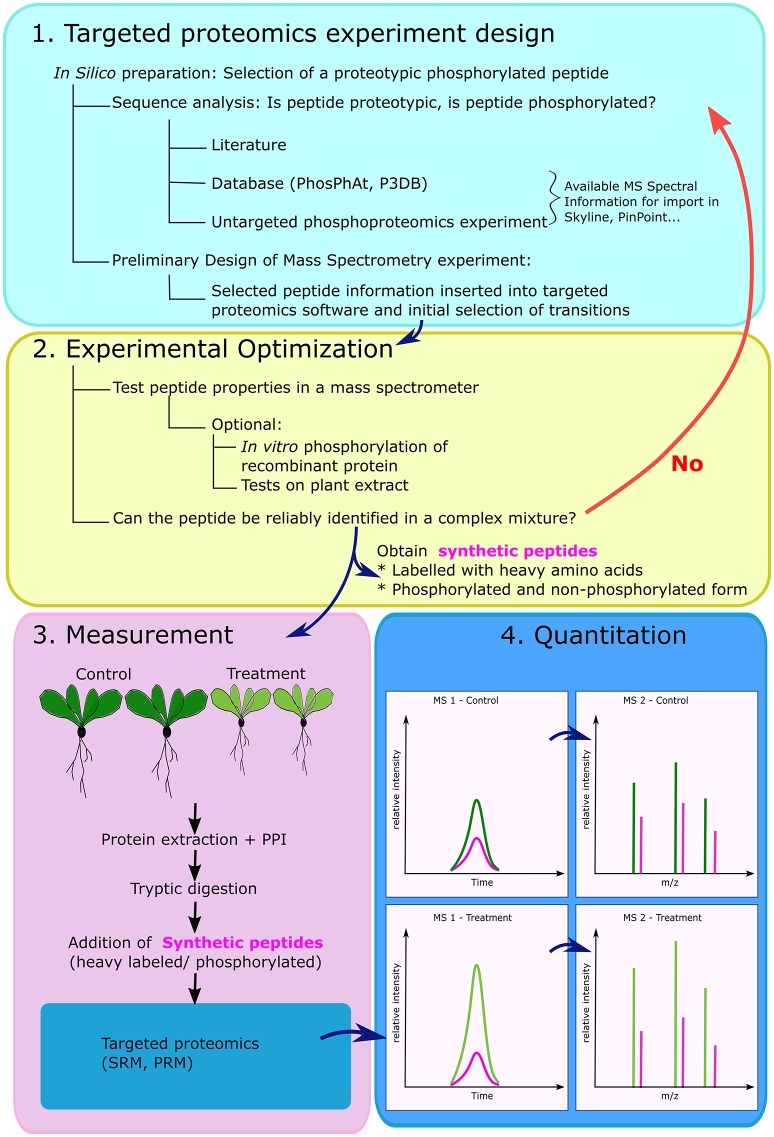
General overview of the steps involved in the creation of a targeted proteomics experiment for monitoring phosphorylated peptides. The experimental design starts with selection of a posttranslationally modified peptide, which needs to meet several criteria: to be proteotypic i.e., unique to a protein, and digestible by a selected protease- usually trypsin. *In silico* fragmentation is performed and targeted transitions are inserted into specific software **(1)**. The peptide properties are verified on the MS platform available to the scientist under conditions relevant for the biological investigation. If confirmed, synthetic peptides can be ordered to serve as internal standards for identification and quantitation **(2)**. After performing the biological experiment, with large number of samples, protein extracts are digested and synthetic peptides in phosphorylated and non-phosphorylated form are spiked in the mixture. Peptides are separated using liquid chromatography and measured on mass spectrometer **(3)**. Readers are asked to remember that quantitation is possible either in MS1 (e.g., Selected Ion Monitoring), or using selected fragments in MS2 (Selected/Multiple Reaction monitoring) **(4)**. PPI- protease and phosphatase inhibitors, SRM- selected reaction monitoring, PRM-parallel reaction monitoring. In **(4)** the colors refer to: control sample peptide (dark green), treated sample peptide (light green) and synthetic peptide (pink).

For scientists interested in a targeted phospho-proteomics approach, one protocol is described by Payne and Huang (2017). Aditionally, in Figure 1, we outline a modified version of the protocol from Aldous et al. (2014). An initial concern in plant proteomics generally, is the protein extraction which to be tailored to the plant material with regard to secondary metabolites, e.g., by addition of Polyvinylpolypyrrolidone (PVPP), and can be later removed in a simple centrifugation step-luckily various plant specific protocols exist, which also include the compulsory addition of reagents that prevent protease and phosphatase action.

The first step of any targeted phospho-proteomics approach is the selection of phospho-peptides and the decision which transitions to record (Figure 1.1). Skyline is a platform-independent, open source software created for the design and analysis of targeted proteomics experiments (MacLean et al., 2010). It is applicable to the study of phosphorylated and acetylated peptides (Schilling et al., 2012), bypassing the necessity of costly vendor specific software. Additionally, publicly-available phospho-proteomics databases can be used for initial phospho-peptide and fragment selection. Among them, PhosPhAt (http://phosphat.uni-hohenheim.de/) hosts phospho-proteomics data from Arabidopsis experiments, and incorporates predicted phosphorylation site, kinases-substrate information, and most importantly, the ability to export Mass-Spectra from actual experiments (Heazlewood et al., 2008; Durek et al., 2010; Zulawski et al., 2013). The database hosts both peptide mass-to-charge (m/z) information, as well as fragment information, which can be used directly in SRM experimental design (Arsova and Schulze, 2012). An interactive network of kinases and their substrates can also be explored in the Plant Protein Phosphorylation database (P3DB, http://www.p3db.org/); this database started as a database focusing on phospho-proteins from crop plants, and today aims to be a phospho-proteomics repository for all plants (Gao et al., 2009; Yao et al., 2014). A detailed manual for new users of PhosPhAt and P3DB is available (Schulze et al., 2015). Other available databases include the Medicago Phospho Protein database (Grimsrud et al., 2010), and the dbPPT (Cheng et al., 2014).

In an ideal case, a deposited peptide spectrum can be directly inserted into a targeted proteomics software (Figure 1.1, Arsova and Schulze, 2012). From this fragmentation spectrum, specific –b and –y fragment-ions can be selected for targeted monitoring. In case of modified peptides, a peptide fragment that corresponds to the modification has to be included. Another important step is the software aided prediction of the collision energy in the mass spectrometer, which will fragment the peptide in such a way that the monitored –b and –y ions are obtained (Wolf-Yadlin et al., 2007).

During practical optimization, theoretical information is verified on the MS platform directly available to the scientist using recombinant proteins and/or plant test material. This involves verifications of parameters like collision energy, instrument dwell time or if necessary selection of alternative fragment ions and can be performed using *in vitro* phosphorylation on recombinant PPCK, and /or testing the selected peptides in a complex plant mixture (Aldous et al., 2014). Once the chromatography and MS conditions are confirmed, heavy standard peptides in phosphorylated and non-phosphorylated form are obtained (Figure 1.2). After this preparatory phase, plants grown under a variety of conditions can be subjected to a standard protein extraction including phosphatase and protease inhibitors. If phosphopeptide enrichment is included, QconCAT proteins can be spiked into the samples before tryptic digestion and the enrichment step (Elschenbroich and Kislinger, 2011). Thus, the standards will have to undergo the digestion and phospho peptide enrichment as well and will (i) control sample to sample variation of the enrichment efficiency, and (ii) serve as internal standards for sample-to-sample normalization and quantification by SRM (Figures 1.3,4).

With the appropriate adaptations to preserving the PTM, and adaptation to the MS experimental design, a similar approach could be possible for plant PTMs as already performed in other species (Schilling et al., 2012; Lamoliatte et al., 2013; Tang et al., 2014).

## Examples of targeted proteomics in the analysis of phosphorylated peptides

Since Glinski and Weckwerth (2005) first monitored a phospho-peptide in a complex sample *in vitro*, the approach has been successfully applied in a number of biological studies (summarized in Table 1). For example, in a study analyzing cold acclimation effects on the Arabidopsis vacuole, changes in phosphorylation status of the tonoplast transporters TMT1, TMT2, and VGT, were linked to increased levels of monosaccharides in the vacuole. Interestingly, the proteins of interest did not change in absolute amount, pointing to the importance in phosphorylation in regulation of transport of carbon metabolites in the vacuole (Schulze et al., 2012). The study is particularly interesting because it confirmed similar response in barley, demonstrating transfer of knowledge between species and the use of conserved phospho-peptides (Nakagami et al., 2010). Another targeted study that used the conserved peptide approach to comparatively monitor phosphorylation in two species (Aldous et al., 2014), showed that the diurnal phosphorylation levels of phosphoenol pyruvate carboxylase (PEPC) from C3 and C4 *Flaveria* species were linked to the day time and night time expressed isoforms of the phosphoenol pyruvate carboxylase protein kinase (PPCK). This, in combination with phylogenetic studies allowed the authors to link kinase amount and activity to evolution of C4 photosynthesis.

**Table 1:** Overview of studies using targeted proteomics for the analysis of plant protein PTMs.

**Species**	**Study characteristics**	**Publication**	**Instrument class**	**Quantified Proteins/Peptides/Transitions p.p**	**Modification**	**Key words**
Arabidopsis	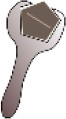	Glinski and Weckwerth, 2005	triple quadrupole	6/16/2	Phosphorylation	*In vitro* phosphorylation, absolute quantitation
Arabidopsis, Barley	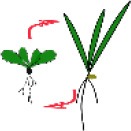 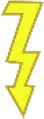 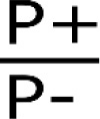	Schulze et al., 2012	triple quadrupole	3/6/minimum 3	Phosphorylation	Cold acclimation
Arabidopsis	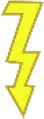 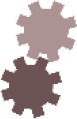	Qiao et al., 2012	triple quadrupole	1/6/1	Phosphorylation	Pseudo-MRM, phosphorylation dependent cleavage and re-location
Arabidopsis	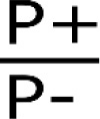	Li et al., 2012	Q-TOF	1/2/0	Phosphorylation	Absolute quantitation on peptide level using metabolic labeling
Arabidopsis	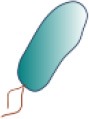 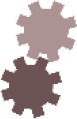	Dubiella et al., 2013	triple quadrupole	1/6/minimum 3	Phosphorylation	Disese resitantce, immunity, ROS
*Arabidopsis*	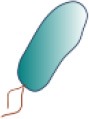 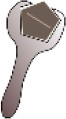	Majovsky et al., 2014	LTQ- Orbitrap	18/48/whole MS/MS spectra	N- End Rule degradation	PRM, degradomics
*Flaveria trinervia, F. pringeli*	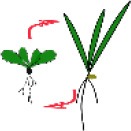 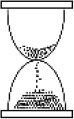 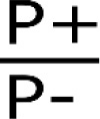	Aldous et al., 2014	triple quadrupole	1/2/3	Phosphorylation	C4 evolution
*Arabidopsis*	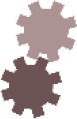 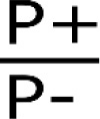	Konert et al., 2015	triple quadrupole	3/3/3	Phosphorylation	ROS signaling, PP2A
*Medicago*	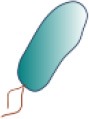 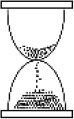	Van Ness et al., 2016	QTRAP	16/16/3–5	Phosphorylation	Symbiotic signaling, TiO_2_
Arabidopsis	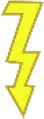 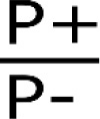	Trotta et al., 2016	triple quadrupole	3/30/3–4	Phosphorylation	Light quality related phosphorylation, link to protein degradation
*Synechocystis* sp.	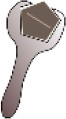	Angeleri et al., 2016	triple quadrupole	19/44/3–19	Phosphorylation	TiO_2_, focus on photosynthetic proteins.
Chlamydomonas	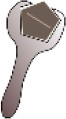	Werth et al., 2017	Triple TOF (Time Of Flight)	1,055 phosphoproteins, 2,250 phosphopeptides	Phosphorylation	Kinome and phopsphoproteome SWATH discovery approach, TiO_2_

*Unless noted otherwise studies use SRM as targeted proteomics technique; p.p, per peptide*. 
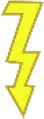
 *Abiotic Stress*, 
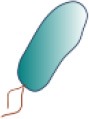
 *Biotic Stress / interaction*, 
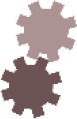
 *Protein - protein Interaction*, 
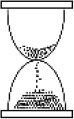
 *Temporal Analysis*, 
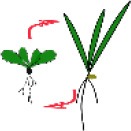
 *Interspecies transfer*, 
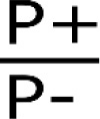
 *Phosphorylation ratio / phospho-site occupancy*, 
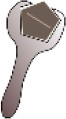
 *Method development*.

Qiao et al. (2012) describes hormone perception and signaling using targeted phosphoproteomics. It links ethylene (a hormone linked to stress and development signaling) to the phosphorylation dependent cleavage and intracellular movement of a C-terminal peptide from the ethylene insensitive 2 protein after a phosphorylation event. The quantification of EIN2 phosphopeptides was carried out using pseudo–MRM, using a triple quadruple instrument (Qiao et al., 2012).

A protein-protein interaction study, describes how the calcium dependent protein kinase-5 (CPK5) drives plant defense mechanism, after reception of a pathogen-associated molecular pattern stimulus. In addition, the action of CPK5 was coupled to a Respiratory Burst Oxidase Homolog (RBOH) by using *in vitro* and *in vivo* SRM, to identify RBOH as a phosphorylation target of the CPK5 (Dubiella et al., 2013). Similarly, the interaction of the Arabidopsis light harvesting complex II (LHCII) which is phosphorylated by the STN7 kinase was described by Trotta et al. (2016). The kinase itself is also subject to differential phosphorylation on its Ser and Thr residues. The results showed that phosphorylation on a Thr residue in the dark, low light, and red light protects the kinase against degradation. Additionally, phosphorylation on the Ser residue occurred only under low and red light, and was linked to the phosphorylation of the LHC II, as target of STN7. By testing multiple light treatments, the authors found that the kinase had three activity states: deactivated in darkness, activated in low and red light, and inhibited by high light (Trotta et al., 2016).

Absolute quantification was demonstrated by Li et al. (2012), by calculating the phospho-site occupancy of a phosphorylated peptide of ERF110 (ethylene response factor 110) by measuring both absolute amount of the whole ERF protein and the amount of phosphorylated peptide around Ser 62 (Li et al., 2012; Yang and Li, 2015). This is one step further from Trotta et al. (2016), and Aldous et al. (2014), who expressed the phosphorylation data in relation to the non-phosphorylated portion of the peptide in the form of a simple ratio.

Time-related phosphorylation dynamic was investigated by Van Ness et al. (2016) during the early time points of Medicago symbiosis establishment. They profiled the period between 5 min and 1 h after bacterial Nod factors were applied to seedlings. The authors used TiO_2_ phosphopeptide enrichment before SRM, and targeted 15 Medicago phospho-proteins. This resulted in the identification of 5 early responding membrane bound phosphoproteins: an SNF1-related kinase, a zinc finger protein, a proton ATP-ase, and two proteins of unknown functions. The authors report that TiO_2_ enrichment increased relative abundance of the phosphorylated peptide and decreased overall sample complexity (Van Ness et al., 2016). Decreasing the sample complexity was also used in the study of protein phosphatase 2A (PP2A) and its interacting partner ACONITASE 3 (Konert et al., 2015). Here, phospho-peptides were first separated by SDS page before undergoing SRM, and the authors report that this process greatly increased the method sensitivity.

The use of targeted proteomics to monitor phosphorylation was also successfully applied to cyanobacteria, to study the phosphorylated photosynthetic proteins in *Synechocystis* sp. (Angeleri et al., 2016). In this example, the targeted approach followed an initial discovery phosphoproteomic study from which 44 phosphopeptides were targeted by SRM. The SRM experimental design alone set the basis for further quantitative studies of this cyanobacterium under various environmental stresses.

## Potential for the use of targeted proteomics in the analysis of other PTMs

Multiple protein PTMs are reported in plants (Friso and van Wijk, 2015; Canut et al., 2016), and their study poses challenges inherent to the chemical nature and abundance of each PTM. Targeted approaches are not yet widely used in the plant field, even though plant acetylation, SUMOylation, and methylation have been studied successfully using discovery proteomics (Deng et al., 2010; Miller et al., 2010; Song and Walley, 2016; Meng et al., 2018), and targeted proteomics on multiple protein PTMs has been performed in other organisms outside the plant kingdom (Schilling et al., 2012; Lamoliatte et al., 2013; Tang et al., 2014)

Similarly, targeted proteomics approaches were established in the human field for the analysis of ubiquitinated proteins (Biel et al., 2004; Mollah et al., 2007), but are still not widely applied in plants. Exceptionally, a parallel reaction monitoring (PRM) approach to examine protein stability using mutants of the N-end degradation rule (i.e., before the proteins are targeted by Ubiquitin ligases) successfully quantified a number of targets of this pathway in *Arabidopsis thaliana* (Majovsky et al., 2014). This demonstrates the potential that targeted proteomics has for the directed study of various PTMs in plant research, as these approaches can be invaluable for physiological/biochemical understanding of protein regulation.

## Conclusions and view ahead

Protein phosphorylation has been predicted for more than 7800 Arabidopsis genes, and each of these PTMs can have an impact on the functional status of the protein. The study of phosphorylated proteins is challenging due to the labile nature and low abundance of the modification. The use of targeted proteomics allows sensitive monitoring on selected phospho-sites, but through a large number of conditions. This is crucial for functional characterization of the protein modification. These measurements can often circumvent phospho-peptide enrichment protocols and can be carried over between species. Plant phospho-peptide databases provide the public with PTM data directly usable for targeted proteomics experiments. Learning from targeted approaches from the non-plant field, analysis of further PTMs are possible, but require further method development in plants.

Increasing the number of reliably and quantitatively monitored PTM peptides in a single study is also a challenge. Recent advances in the human field have optimized standard PRM protocols and precise quantitation of 600 peptides can be performed (Gallien et al., 2015). An exceptional study from the human field used more than 18,000 recombinant proteins as internal standards for precise quantitation on a genome-wide scale; demonstrating the full potential of targeted proteomics (Matsumoto et al., 2017). One must consider that development of similar platforms for the monitoring of PTMs will be more complicated, as all these peptides would have to be correctly modified *in vitro*. Notably, similar coverage to Matsumoto et al. (2017) could also be achieved with the Sequential Windowed Acquisition of All Theoretical Fragment Ion Mass Spectra (SWATH MS) approach (Gillet et al., 2012), examples of which use are emerging in green biology e.g., for *Chlamydomonas* (Werth et al., 2017). We expect an increase of these approaches in the near future facilitated by improvement of data-analysis software, and bringing closer untargeted and targeted proteomics. However, the major strengths: absolute quantitation and high throughput possibilities must be kept to allow the move from relative to absolute quantification.

## Author contributions

BA conceived the idea, wrote the initial manuscript and made the figure, MW revised the manuscript and contributed with references, BU performed major revisions and contributed with valuable discussions.

### Conflict of interest statement

The authors declare that the research was conducted in the absence of any commercial or financial relationships that could be construed as a potential conflict of interest.
